# Dietary Patterns of Competitive Swimmers with Moderate-to-Severe Cerebral Palsy: A 3-Year Longitudinal Evaluation

**DOI:** 10.3390/ijerph20075331

**Published:** 2023-03-30

**Authors:** Jacqueline L. Walker, Jessica R. Cartwright, Iain M. Dutia, Mikaela Wheeler, Sean M. Tweedy

**Affiliations:** School of Human Movement and Nutrition Sciences, The University of Queensland, St Lucia, QLD 4072, Australia

**Keywords:** dietary patterns, performance-focused, swimming training intervention, cerebral palsy, adolescents

## Abstract

Aim: To evaluate the longitudinal dietary patterns of three adolescents with moderate-to-severe cerebral palsy (CP) participating in a performance-focused swimming training intervention. Method: Participants were three previously inactive adolescents with CP (15–16 years, GMFCS IV) who had recently (<6 months) enrolled in a swimming training program. Diet quality from diet histories was calculated at 10-time points over 3.25 years using the Dietary Guidelines Index for Children and Adolescents (DGI-CA) and the Healthy Eating Index for Australian Adults (HEIFA-2013). A food group analysis was compared to the Australian Guide to Healthy Eating recommendations. Trends were considered in the context of dietary advice given and the training load. Results: Longitudinal diet quality scores were consistent and ranged from 40 to 76 (DGI-CA) and 33 to 79 (HEIFA-2013). Food group intake remained stable; participants rarely met the recommendations for fruit, vegetables, dairy, grain, and meat but frequently achieved discretionary serves. Conclusions: Participants with moderate-to-severe CP who were enrolled in a performance-focused swimming training intervention and were monitored frequently maintained diet quality throughout a period where it conventionally declined. Scores were higher than the general population and were maintained irrespective of the training load. Participants frequently met food group recommendations for discretionary foods and were comparable to the general population for other food groups.

## 1. Introduction

Cerebral palsy (CP) is an umbrella term for a heterogenous cluster of neurological disorders caused by static and permanent injury to the developing foetal or infant brain [1]. Impaired motor function is the defining feature of CP, which often presents with reduced strength, changes in muscle tone, a decreased range of motion, and skeletal deformity [1]. Approximately 35% of people with CP have high support needs [2]. These individuals are at Gross Motor Function Classification System (GMFCS) level IV or V, cannot mobilise independently, and require physical assistance for one or more functional tasks of daily living [3].

People with CP are less physically active than the general population, and those with moderate-to-severe CP are the least physically active subset of the population [4]. Consequently, this group experiences high morbidity and is at increased risk of mortality [4]. The ParaSTART (Sports Training and Research Team) program is a university-based training program that addresses this problem by supporting young people with CP to be physically active through sports. A unique program feature is a focus on competitive sports performance—ParaSTART aims to help people with CP pursue sporting excellence, as opposed to facilitating participation for clinical, health, or recreation purposes [5]. Underpinning ParaSTART is a program of research that focuses on outcomes related to sports performance, which can be broadly classified into topic areas of performance, physical activity, nutrition, and psychosocial function [5].

In adolescents with CP, nutrition concerns are common [6]. Factors such as inadequate dietary intake contribute significantly to nutritional concerns, which have been linked to suboptimal growth, cerebral dysfunction, decreased muscle strength, decreased societal participation, and decreased general well-being [7,8]. Significant examples in the literature exist to show that motor function can be a predictor of nutrition outcomes [6,7]. Children and adolescents with moderate-to-severe CP consistently display poorer nutritional outcomes compared to those living with mild CP [6,7,8]. This highlights the importance of adequate nutrition in a non-ambulant CP population.

There is a body of literature that explores longitudinal changes in the nutrition of infants and children with CP due to the interest in early intervention [9]. Meanwhile, adolescence, as another key developmental stage, is largely unexplored. Existing long-term studies focus on health and clinical outcomes such as bone mineral density or the effect of gastrostomy devices, which arise from various clinical interventions [10,11]. In the context of the ParaSTART Program, it is paramount that nutritional intake (encompassing energy, macronutrient, micronutrient, and fluid intake) adapts over time to reflect changes in physical activity levels and energy expenditure, as optimal performance is realised only when nutrition is managed in conjunction with physical activity [12]. Despite this, the literature exploring dietary factors in adolescents with CP training for sports performance remains scarce. The most comparable study assessed dietary intake and quality in para-athletes over four consecutive training days [13]. Participants, however, were a group of adults living with varying disabilities; therefore, caution must be taken when generalising results to a CP population [13].

Furthermore, broader nutrition measures that emphasise healthy eating and diet quality have not been investigated thoroughly in this population. As stated previously, studies focus on clinical factors such as micronutrient deficiencies, feeding routes, food textures, and nutritional requirements that are usually reported cross-sectionally [6,7,8]. Encouragingly, a 2020 study conducted by Williams and colleagues focused on adolescents with moderate-to-severe CP and assessed dietary patterns using the Modified Child Nutrition Questionnaire [14]. Of the 12 participants, only 9% managed to achieve all nine nutrition targets that aligned with national healthy eating guidelines [14]. This study, however, was cross-sectional in nature and lacked a sports focus.

It is important to understand nutrition outcomes in the context of physical activity and sport, as opposed to traditional clinical interventions. By focusing on dietary patterns, which are the quantities, proportions, variety, or combination of different foods, drinks, and nutrients in diets, and the frequency with which they are habitually consumed [15], researchers will gain insights that are more pragmatic in nature and have the potential to be modified in the future. The aim, therefore, of the current study was to determine and understand the longitudinal dietary patterns of adolescents with moderate-to-severe CP participating in a performance-focused swimming training intervention.

## 2. Materials and Methods

### 2.1. Participants

Participants in this study were taking part in The ParaSTART Program at The University of Queensland, Brisbane, Australia. Details regarding the methods of recruitment can be found in the associated protocol paper [5]. At program commencement, the participants were classified as GMFCS III, IV, or V, water safe but unable to swim for fitness, aged 15–35 years old, and not meeting physical activity guidelines [5]. Parents/guardians provided informed, written consent for all participants under the age of 18 years. In addition, written assent was obtained from all participants under the age of 18 years. All participants had an adequate cognitive function to provide their own assent as relevant for their age.

### 2.2. Study Design and Intervention

**Figure 1:**
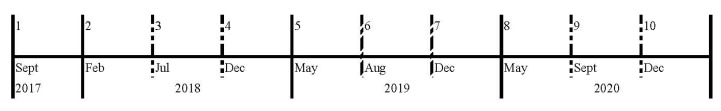
The 10 study assessment timepoints from 2017 to 2020.

The current paper reports on the observational, longitudinal findings regarding nutrition-related data. It stemmed from the ParaSTART Program, which used multiple-baseline, single-case experimental design (SCED) methodology to evaluate, inter alia, the clinical responses of people with CP to performance-focused swimming training. The SCED study was carried out over 16 months, between March 2017 and July 2018, and the methods used are described elsewhere [5]. Upon completion of the SCED study, the training program was continued, and the study period was extended to four years. The sole aim of all strategies employed in the training intervention was to improve competitive swimming performance over 50 metres. The training was delivered by a multi-professional team of qualified physiotherapists, exercise physiologists, and swim coaches. This paper reports the changes in dietary behaviour that occurred between September 2017 and December 2020—representing three years and three months (3.25 years) of the four-year study period. The ten study assessment time points are shown in Figure 1. During the first year of the study, participants received individualised nutrition support from a final-year Master of Dietetics student. The intensity of this support was dependent upon participant need, but all advice given focused on improving nutritional intake from a sporting perspective and was in alignment with the Australian Guide to Healthy Eating (AGHE) [16]. After this period, participants or their parent/carer could request further dietary advice as required.

### 2.3. Data Collection

Nutritional data were collected prospectively by a research dietitian over 10-time points from September 2017 to December 2020, approximately three times per year, aligning with particular training periods. The nutritional data collected included diet histories which were completed by the research dietitian in liaison with the participant and their guardian/s using an adapted version of the Diet History Questionnaire [17], along with food models and measuring cups/spoons/bowls/plates to obtain a high level of detail and accuracy. Data were collected in paper form, then scanned and stored on a database management system. The outcome measures included diet quality and food group serves. The training load was included as a process measure of physical activity dose, comprising the frequency, intensity, and duration of training [5].

### 2.4. Data Analysis

Diet quality was determined for each timepoint for all participants by calculating both the Dietary Guideline Index for Children and Adolescents (DGI-CA) score and the Healthy Eating Index for Australian Adults (HEIFA-2013) score from the collected diet history data. The DGI-CA tool has been validated for use in children [18], and it uses 11 indicators to reflect the updated 2013 Australian Dietary Guidelines and produce a score out of 100 [18]. Higher scores reflect greater adherence to the dietary guidelines, as it is indicative of diet adequacy, quality, variety, and moderation [18]. During the data collection period, the participants aged from approximately 17 to 20 years old; thus, it was inappropriate to solely rely on the DGI-CA to inform diet interpretation. To overcome this, the HEIFA-2013 was also used to calculate diet quality scores for all participants, as it has been validated for use in individuals aged 18 years and above [19]. Similar to the DGI-CA, it used 11 indicators reflecting the 2013 Australian Dietary Guidelines to produce a score out of 100 and is a useful tool to monitor changes in the dietary intake of adults over time [19]. Analysis using both tools allowed for greater accuracy and interpretation of data. One researcher that had the required background knowledge, training, and experience used the diet quality tools to code and interpret diet history data from the 30 data points (10 per participant) so that the final diet quality scores could be determined. Intra-rater reliability was established via a Bland and Altman analysis [20] using a randomised sub-sample of data points. A concurrent food group analysis was conducted, which involved comparing food group serves to the AGHE recommendations for the corresponding age and gender of each participant [16]. For discretionary foods, recommended serves were based on the DGI-CA [18] and HEIFA-2013 [19] indexes, as the AGHE [16] does not specify discretionary serves. Descriptive statistics were used to describe the DGI-CA [18] and HEIFA-2013 [19] scores amongst participants. The trends observed in diet quality were considered in the context of the dietary advice given, the training schedule, and the load (data previously collected in the overarching study). All data analyses used each participant as their own control to observe changes over time.

### 2.5. Ethics

Ethics approval was granted by the Human Research Ethics Committee of The University of Queensland (approval number 2018001472).

## 3. Results

Three adolescents were involved in the study. As shown in Table 1, at enrolment, participant 1 (P1), participant 2 (P2), and participant 3 (P3) were at GMFCS level IV and had efficient eating and drinking abilities with minor limitations [21].

### 3.1. Reliability

**Figure 2:**
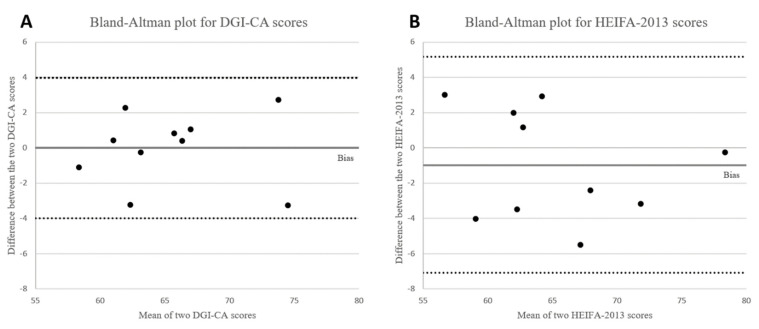
(**A**) Bland–Altman scatter plot for intra-rater reliability scores of the Dietary Guidelines Index for Children and Adolescents (DGI-CA) and (**B**) the Healthy Eating Index for Australian Adults (HEIFA-2013).

A subsample of 10 data points (30% of all data points) was analysed twice, approximately three weeks apart, to determine intra-rater reliability for both DGI-CA and HEIFA-2013 measures. Reliability was established at a 0.03% (DGI-CA) and a 1.4% (HEIFA-2013) difference in the scores overall. The standard deviation of differences was 2.03 and 3.12, and limits of agreement ranged from −3.99 to 3.97 and −7.10 to 5.15 for DGI-CA and HEIFA-2013, respectively. A visual inspection of the plots in Figure 2 shows there are no points that lie outside these ranges, and they are within close proximity to the bias line. The range between the limits of agreement is smaller in DGI- CA than it is for the HEIFA-2013, indicating better agreement.

### 3.2. Diet Quality

**Figure 3:**
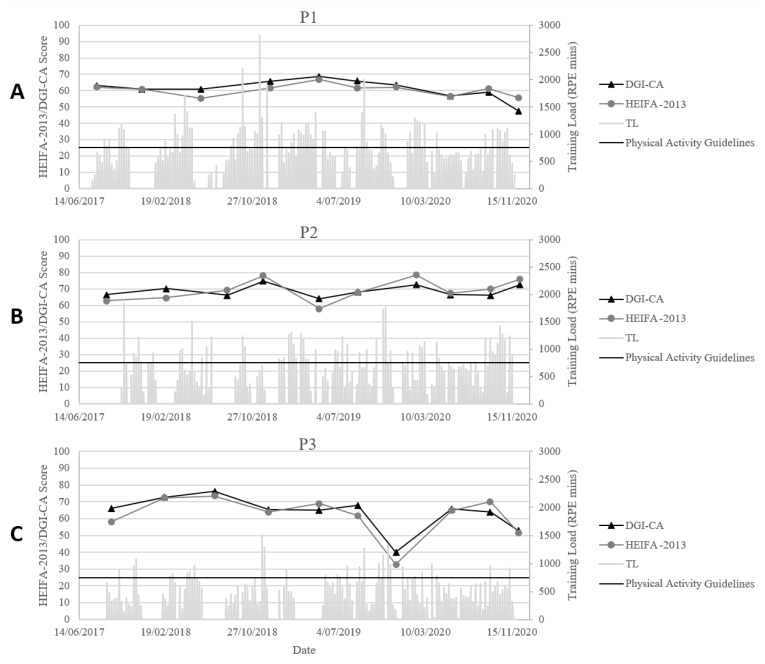
Training load (TL) data (bar graph) and the associated physical activity recommendation for the general population (red line) overlayed with the dietary index scores (line charts)—Dietary Guideline Index for Children and Adolescents (DGI-CA), and the Healthy Eating Index for Adults (HEIFA-2013) for participant 1 (P1) (**A**), participant 2 (P2) (**B**), and participant 3 (P3) (**C**).

Figure 3 displays the DGI-CA and HEIFA-2013 scores for participants across time points. The DGI- CA scores ranged from 47 to 69 (x ± SD = 61 ± 6) for P1, 64 to 75 (69 ± 4) for P2, and 40 to 76 (64 ± 10) for P3. The HEIFA-2013 scores ranged from 55 to 67 for P1 (60 ± 4), 58 to 79 for P2 (69 ± 7), and 33 to 73 for P3 (62 ± 12). As Figure 3 shows, at timepoint 7 for P3, there was a drop in both scores; however, this participant rectified their score by the next assessment point. Figure 3 also shows no clear upward or downward trend, which highlights the consistency in scores over the 3.25-year timeframe.

### 3.3. Training Load

The training load data over the experimental period are also displayed in Figure 3. This was individualised and, therefore, different for each participant. The training load for P1 met or exceeded the physical activity guideline equivalent of 750 Rate of Perceived Exertion (RPE) minutes [22] in 41% of the training weeks. P1 achieved greater than half of the physical activity guideline load [22] in 74% of the training weeks. The training load for P2 met or exceeded the physical activity guidelines [22] in 16% of the training weeks, and P2 achieved greater than half of the physical activity guideline load [22] in 67% of the training weeks. The training load for P3 met or exceeded the physical activity guideline [22] in 28% of the training weeks, and P3 achieved greater than half of the physical activity guideline load [22] in 80% of the training weeks.

### 3.4. Food Group Analysis

Table 2 summarises if and when participants met the food group recommendations according to AGHE [16]. P3 was the only participant to meet recommended fruit serves and did so at three adolescent time points. P1 had no fruit intake for eight of the 10-time points and P2 averaged approximately 0.7 serves/day. No participant met the vegetable recommendations at any time point, nor achieved over three serves of vegetables/per day. P2 had the highest vegetable intake—consuming 2.6 serves on three occasions. All participants achieved the recommendations for grains at least once. P1 had a minimum of 3.5 serves/day, and P2 and P3 had a minimum of 2.5 serves/day. Each participant consumed dairy products daily. The lowest dairy intake for P1 was approximately 0.5 serves/day, and approximately 1.3 serves/day for P2 and P3. The lean meat intake recommendations were more regularly achieved by participants. Throughout the 10 timepoints, P1 averaged 2.2 serves of meat/day, P2 consumed 2.8 serves/day, and P3 consumed 2 serves/day. The discretionary recommendation was the most attained of all food groups, as P2 met the requirements on all occasions (averaging 1.2 serves/day), P1 on all but one occasion (averaging 1.7 serves/day), and P3 on five out of 10-time points (averaging 2.3 serves/day). Overall, there was no clear improvement or regression over 3.25 years to achieve any of the food group recommendations.

## 4. Discussion

This study aimed to explore longitudinal dietary patterns of individuals with moderate-to-severe CP participating in a swimming training intervention during adolescence and young adulthood. Though it could be reasonably expected that a decrease in diet quality could be observed over the project timeframe, the scores for participants were maintained. The determined reliability bias was minimal, enabling a meaningful interpretation of the DGI-CA and HEIFA-2013 scores. At the time of enrolment, the participants had a high dietary quality, which was likely to have been reinforced by individualised dietary advice and support over the first year. Furthermore, the data collection time points acted as a ‘check in’ for dietary monitoring and maintenance and offered a time when participants could reflect with the research dietitian on their current intake. These aspects support the maintenance of positive dietary behaviour changes and, theoretically, ensure that participants are less likely to experience a dietary decline over time [23]. Despite intense training loads for all participants, Figure 3 suggests that the diet quality was not impacted. Participants demonstrated a long-term adoption of physically active behaviour through sustained engagement in the training over a prolonged period: an achievement rarely demonstrated previously in the literature in people with moderate-to-severe CP. This positive outcome adds a unique element when interpreting the dietary data.

DGI-CA and HEIFA-2013 scores for participants over time were consistently comparable to the general population [18,24]. In an Australian study by Golley et al., the mean DGI-CA score for 14- to 18-year-olds was 48 [18]. The participants in the current study had a mean DGI-CA score of 61 (P1), 69 (P2), and 64 (P3). The mean HEIFA-2013 score for young adults aged from 18 to 24 years in a study by Grech and colleagues was 42, whereas the average scores for P1, P2, and P3 were 60, 69, and 62, respectively [24]. This highlights that participants overall had a better diet quality than the general public, which is extremely encouraging, considering the known challenges of obtaining and maintaining an optimal dietary intake for people with moderate-to-severe CP [6,7,8]. A cross-sectional study of adult sprinters within the Brazilian Paralympic track and field team used a comparable dietary index tool and also found their para-athletes (20% of whom had mild-to-moderate CP) to have scored higher than the general public [13]. The tool used by Joaquim and colleagues was a Healthy Eating Index based on the Brazilian Dietary Guidelines [13]. The results of the current study, therefore, are extremely promising, considering that participants were novice athletes of adolescent age. The individual variation in scores for both indexes for all participants was within 20 points (with the exclusion of the timepoint 7 outlier of P3) which suggests a high consistency in diet quality over time. Timepoint 7 for P3 can be considered an anomaly, as the participant was recovering from surgery and experiencing poor oral intake coupled with weight loss. Moreover, this coincided with the end of the school year and the start of the festive season, which may be contributing factors. By timepoint 8, however, the diet quality was rectified and consistent with previous scores. The maintenance of diet quality for all participants whilst undergoing a transition from adolescence to adulthood is encouraging, even when taking into account the data collection ‘check ins’. The existing literature suggests that when students transition at the end of their schooling, there can be a decrease in diet quality [25,26,27,28]; however, this shift in autonomy has not affected diet quality in our participants. This could be explained by several factors, including personal qualities, motivating interests, program participation, and supportive family members, details of which need to be elucidated in further studies.

With regard to the food group analysis, findings are comparable to the general population for the five food groups, with discretionary serves for the participants being much lower [16,18,29]. According to the Australian Bureau of Statistics data from 2018, Australians of all ages did not consume enough of the five food groups and ate too many discretionary foods [29]. The first part of this statement aligns with the present findings, as the fruit, vegetable, grain, dairy, and lean meat recommendations were infrequently achieved. These food groups provide essential macro and micronutrients for optimal growth and development and are even more important when considering the additional demands of sports training and performance. For example, an adequate intake of protein is needed for muscle repair and rebuilding; therefore, the role of dairy products and strategies to increase an intake in various situations (pre-training, competition, recovery) would be an essential component of a nutritional intervention following these study findings. The main difference between the study population versus the general population involved an intake of discretionary items. For typically developing adolescents aged from 14 to 18 years, recent literature confirms that 41% of energy intake comes from discretionary foods, equating to approximately 4.1 serves/day [16,18]. Dissimilarly, participants in the current study consumed less than half of this. In adults, 5–7 serves of discretionary foods were consumed each day [29]. Once the participants in this study transitioned into adulthood, their discretionary serves were roughly one-quarter of that of the general population. In clinical settings, it is often perceived that it is difficult and unlikely for people with moderate-to-severe CP (even those with a lower Eating and Drinking Classification System (EDACS) score [21]) to meet national dietary recommendations. The current findings challenge this notion, showing that the national dietary guidelines are still applicable and achievable, and over the data collection period, there were no negative trends that transpired, again implying longitudinal diet maintenance. Using the national dietary guidelines as targets during a follow-up nutritional intervention with this population would be appropriate prior to applying targets that are more applicable to high-performance sporting situations (for example, dietary protein intake in grams per kilogram per day). This is likely to be more practical and achievable for participants, using foods and fluids that are familiar to them.

Despite attempts to account for methodological drawbacks, there are inevitable limitations. This study only had three participants with moderate-to-severe CP; however, these were classified as EDACS level I or II and were highly motivated volunteers, so caution should be taken when extrapolating these findings to varying CP populations. There were also limitations regarding the use of dietary index tools. The DGI-CA was validated using a 48 h diet recall, and HEIFA-2013 was validated using a weighed food record and food frequency questionnaire. This study used a diet history, which required further data interpretation prior to analysis. A strength was that the analysis was conducted by one trained, knowledgeable, and experienced researcher, meaning all assumptions and interpretations were consistent across participants and time points, removing any potential opportunity for discrepancies to arise.

## 5. Conclusions

This study is the first of its kind, aiming to evaluate the longitudinal dietary patterns of adolescents with moderate-to-severe CP participating in a performance-focused swimming training intervention. Participants demonstrated long-term diet quality maintenance, enhanced diet quality, and more favourable discretionary food intake compared to the general population. The results indicate that for non-ambulant young people with CP who have a low EDACS score, the AGHE recommended food serves are applicable and achievable. Future research is warranted to evaluate the long-term dietary patterns in populations of people with CP to support these insights and strengthen links between enhanced diet quality, para-sports, and the impact on performance.

## Figures and Tables

**Table 1:** Participant characteristics at commencement of the ParaSTART Program.

Participant	Gender	Age	Motor Type/Distribution	GMFCS	EDACS
P1	Male	15 yr, 2 mo	Spastic Quadriplegia	IV	I
P2	Male	15 yr, 7 mo	Spastic Quadriplegia	IV	II
P3	Female	15 yr, 7 mo	Choreoathetosis	IV	I

EDACS = Eating and Drinking Ability Classification System, GMFCS = Gross Motor Function Classification System, P1 = Participant 1, P2 = Participant 2, P3 = Participant 3.

**Table 2:** Food group analysis for each participant over the data collection period by comparing adolescent data points to national adolescent recommendations, and adult data points to national adult recommendations.

Food Groups		Recommended Serves *		Timepoint									
		14–18 y/o	19–50 y/o	1	2	3	4	5	6	7	8	9	10
				Sept’17	Feb’18	Aug’18	Nov’18	May’19	Aug’19	Feb’20	May’20	Sept’20	Dec’20
Fruit	P1	2	2										
	P2	2	2										
	P3	2	2	√	√		√						
Vegetable	P1	5.5	6										
	P2	5.5	6										
	P3	5	5										
Grain	P1	7	6		√			√					
	P2	7	6				√		√	√		√	
	P3	7	6				√						
Dairy	P1	3.5	2.5		√		√						
	P2	3.5	2.5						√				
	P3	3.5	2.5			√							
Meat	P1	2.5	3					√	√	√			
	P2	2.5	3		√	√	√	√	√	√		√	
	P3	2.5	2.5		√	√					√	√	
Discretionary	P1	2	3	√	√	√	√	√	√	√	√	√	
	P2	2	3	√	√	√	√	√	√	√	√	√	√
	P3	2	2.5			√			√		√	√	√

P1 = participant 1, P2 = participant 2, P3 = participant 3, white cell under Timepoints 1 to 10 = participant under adolescent recommendations, grey cell under Timepoints 1 to 10 = participant under adult recommendations, √ = recommendation met, blank = recommendation not met, * = Recommended serves as per the Australian Guide to Healthy Eating with the exception of the discretionary foods group, where the recommendation from either the Dietary Guidelines Index for Children and Adolescents (DGI-CA) or Healthy Eating Index for Australian Adults (HEIFA-2013) was used.

## Data Availability

The data that support the findings of this study are available on request from the corresponding author (J.L.W.). The data are not publicly available due to their containing information that they could compromise the privacy of research participants.
